# Efficacy and safety of Shufeng Jiedu Capsule in the treatment of acute exacerbations of chronic obstructive pulmonary disease: A protocol for systematic review and meta-analysis

**DOI:** 10.1097/MD.0000000000024198

**Published:** 2021-01-08

**Authors:** Huijun Ren, Yuhao Jiang, Shiyu Wang, Yirong Wang, Jingying Wang

**Affiliations:** a School of Pharmacy, Chengdu University of Traditional Chinese medicine; b Chengdu Third People's Hospital.

**Keywords:** acute exacerbations of chronic obstructive pulmonary disease, chronic obstructive pulmonary disease, protocol, Shufeng Jiedu, systematic review, traditional Chinese medicine

## Abstract

**Background:**

With the outbreak of novel coronavirus, the treatment of respiratory diseases has been promoted. In particular, many traditional Chinese medicines, including Chinese patent medicines, have been found to be effective in the treatment of respiratory illness in China. chronic obstructive pulmonary disease (COPD) is one of most common respiratory condition. It is predicted that COPD will be become the third frequent cause of death by 2030. The aim of this study is to assess the efficacy and safety of Shufeng Jiedu Capsule in the treatment of acute exacerbations of chronic obstructive pulmonary disease (AECOPD).

**Methods:**

According to the search strategy, randomized controlled trials (RCTs) of Shufeng Jiedu Capsule in the treatment of AECOPD were obtained from Cochrane Library, MEDLINE, Embase, CNKI, VIP, CBM, and WANGFANG. Studies were screened according to inclusion and exclusion criteria, and the Cochrane risk bias assessment tool was used to assess the quality of the study. Meta-analysis was performed using Revman 5.4 software. Finally, the evidence level of the results will be evaluated.

**Results:**

The purpose of this study was to evaluate the efficacy and safety of Shufeng Jiedu Capsule in the treatment of AECOPD, and to provide basis for clinical rational drug use.

**Conclusion:**

Our research results of this study could provide reference for clinical decision-making and guiding development in the future COPD patient.

**INPLASY registration number:**

INPLASY2020120062

## 1 Introduction

Chronic obstructive pulmonary disease (COPD) is a common and progressive, chronic lung disease, which is characterized by persistent respiratory symptoms and an enhanced chronic inflammatory response in the airways.^[1]^ The World Health Organization has predicted that COPD will become the third frequent cause of death until 2030,^[2]^ and the Global Burden of Disease (GBD) study shows that it was the third main cause of death worldwide in 2016 by about 3.0 million deaths.^[3]^ The main risk factors of COPD are smoking, second-hand smoke, air pollution and exposure to fuel fumes.^[4]^ COPD is diagnosed on the basis of symptoms such as dyspnea (shortness of breath), cough or expectoration (or both), and persistent airflow limitation is confirmed by vital capacity measurement.^[5]^ Dyspnea is the most common symptom reported by COPD patients and is associated with deterioration of their quality of life and physical activity. With the development of the disease, dyspnea will usually aggravate. The severity of the disease is associated with the frequency of deterioration or “sudden onset” and the presence of other comorbidities, such as cardiovascular disease, musculoskeletal injury, or diabetes.^[6]^ Despite optimisation of treatments, some patients with COPD continue to experience debilitating symptoms that can impact on their functional status and quality of life (e.g.increased exacerbations, hospitalisations, and risk of mortality).^[7]^

The aim of current routine pharmacotherapy for stable COPD is to control disease progression by using bronchodilators and anti-inflammatory drugs.^[8]^ But, a wind variety of factors may lead to the acute exacerbation of COPD (AECOPD). The most common causes are respiratory tract infections caused by bacteria or viruses (possibly coexisting), and non-infectious environmental factors such as pollution or allergens. In the treatment of AECOPD, bronchodilators are usually combined with systemic corticosteroids, antibiotics and other respiratory support.

However, the treatment outcomes remain less than satisfactory and these interventions are known to cause a variety of adverse effects, including, cardiovascular events, oropharyngeal candidiasis and risk of pneumonia.^[8]^ In China, the use of the complementary and alternative medicine (CAM) is relatively common with COPD sufferers. And Chinese medicine has become increasingly accepted worldwide.^[9]^

Shufeng Jiedu (SFJD) capsule, which is an oral patent Chinese herbal medicine, used widely in China for the treatment of respiratory disease. Eight medicinal herbs make up this capsule, which contains Rhizoma Polygoni Cuspidati, Fructus Forsythiae, Radix Isatidis, Radix Bupleuri, Herba Patriniae, Herba Verbenae, Rhizoma Phragmitis, and Radix Glycyrrhizae.^[10]^

Recently, the basic research on Shufeng Jiedu Capsule shows that it can improve the lung function and reduce the inflammatory index by anti-inflammatory, immunomodulating and antiviral properties,^[10–13]^ which has the theoretical basis for the treatment of respiratory diseases. And combination of Chinese medicine and western medicine can improve the clinical symptoms and quality of life better than the western medicine alone.^[14–26]^ Some reviews have published about the effectiveness and safety of Shufeng Jiedu Capsule in the treatment of respiratory disease, even COPD. However, there are many new RCTs recently, which have not been included in the previous systematic evaluation. It is necessary to reevaluate its efficacy and safety. Therefore, our purpose is to collect the latest information of Shufeng Jiedu Capsule for AECOPD and evaluate the therapeutic effect and safety, and provide help for clinical decision-making.

## 2 Methods and analysis

### 2.1 Objectives and registration

This review will be to assess the efficacy and safety of Shufeng Jiedu Capsule for AECOPD. The protocol for this systematic review has been registered on the International Platform of Registered Systematic Review and Meta-Analysis Protocols (INPLASY). The registration number was INPLASY2020120062. And the article will adhere to the Preferred Reporting Items for Systematic Reviews and Meta-Analysis Statement (PRISMA-P reporting guidelines).^[27]^

### 2.2 Data sources and retrieval strategy

Studies were obtained from the PubMed, Embase and Cochrane Library, China National Knowledge Infrastructure, Wan Fang Data, Chinese Scientific Journals Database, regardless of publication date or language.

The databases were searched by combining the subject words with random words. The retrieval strategy is shown in Table 1 using PubMed retrieval as an example. The search terms were adapted appropriately to conform to different syntax rules of different databases.

**Table 1:** Retrieval strategy of PubMed. Search strategy used in PubMed database.

Number	Search Term
1	“AECOPD” [MeSH] OR “acute exacerbations of chronic obstructive pulmonary disease” [Title/Abstract] OR “COPD” [Title/Abstract] OR “chronic obstructive pulmonary disease” [Title/Abstract]
2	“Shufeng Jiedu Capsule” [Title/Abstract] OR “Shufeng detoxification capsule” [Title/Abstract].
3	Randomized controlled trial [Title/Abstract] OR Controlled clinical trial [Title/Abstract].
4	1AND 2AND 3

### 2.3 Eligibility criteria

The PICOS principles were given full consideration to establish the inclusion and exclusion criteria of this systematic review.

#### 2.3.1 Types of studies

Randomized controlled trials (RCTs) will be included in this systematic review, regardless of publication date or language. Quasi-randomized controlled trials (QRCTs), nonrandomized studies, animal trials, summaries of personal experience and crossover studies will be excluded.

#### 2.3.2 Types of participants

All the patients who have been diagnosed with AECOPD will be included, regardless of their age, gender, or race.

#### 2.3.3 Patient and public involvement

In this study, there is no patient and public involvement in consideration of this protocol for a systematic review.

#### 2.3.4 Types of interventions and comparators

SFJD (capsules, granules, or other types) alone or paired with other routine western medicine will be included. There is no limitation regarding the place of origin, dosage form, dosage, frequency and duration of treatment. The comparisons will be either with other therapeutic agents, or without other treatment or placebo based on conventional treatment of western medicine.

### 2.4 Type of outcomes

#### 2.4.1 Primary outcomes

(1) total efficacy
(2) the number of patients who had any adverse events at the end of treatment

#### 2.4.2 Secondary outcomes

(1) Health-related quality of life
(2) All-cause mortality
(3) length of hospital stay
(4) time to resolution of clinical symptoms

If additional outcomes are reported in the eligible study, these results will be extracted and reported.

### 2.5 Study selection and data extraction

**Figure 1:**
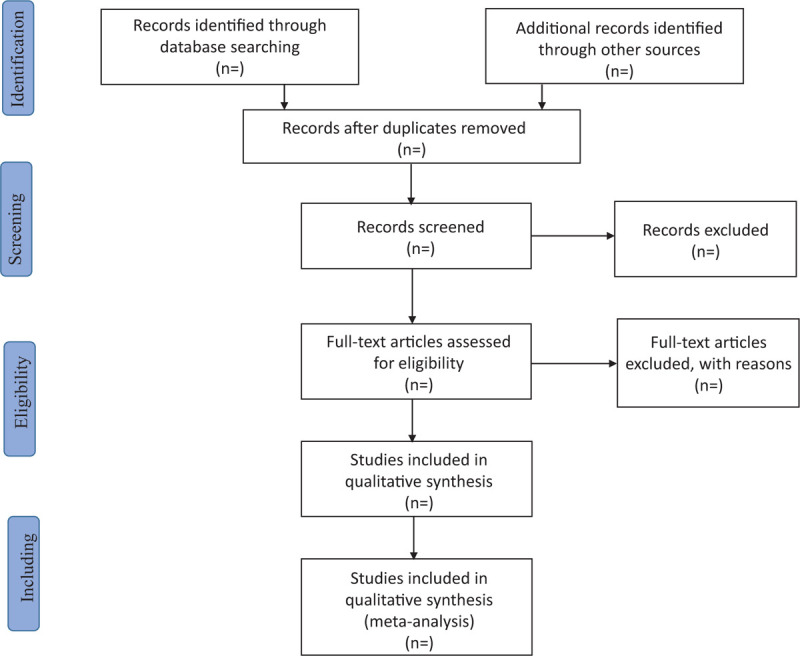
Flow chart of study selection.

EndNote X9 was used to manage the retrieved studies. As shown in Figure 1, the study selection was divided into 2 steps, which were completed by 2 researchers (Yuhao Jiang and Huijun Ren). Preliminary screening included the elimination of duplicate and unqualified studies by reading the title and abstract. Rescreening included reading the full text and selecting studies according to inclusion and exclusion criteria.

According to the Cochrane Handbook for Systematic Reviews of Intervention, 2 researchers (Yirong Wang and Huijun Ren) extracted the author, publication time, number of participants, age, gender, intervention measures, course / treatment and outcome indicators, filled in the data extraction table and compared them with each other.

### 2.6 Risk of bias assessment

Two researchers (Jingying Wang and Yirong Wang) assessed the quality of the included RCTs independently by utilizing the Cochrane risk of bias assessment tool. As specified by Cochrane Handbook V.5.1.0, the following sources of bias were considered: random sequence generation, allocation concealment, participant blinding, outcome assessor blinding, incomplete outcome data, selective reporting, and other sources of bias. Each domain was rated as having a high, low or unclear risk of bias as appropriate.^[28]^ The 2 reviewers resolved any disagreements through discussion, and a third reviewer (Huijun Ren) was involved if a consensus could not be reached.

### 2.7 Statistical analysis

The meta-analysis was performed with Review Manager 5.4 software. The outcomes were mainly represented by the mean difference (MD) or odds ratio (OR) with 95% confidence intervals, and a P value <.05 was considered significant. The Cochrane *Q*-test and *I*2 statistics were used to assess heterogeneity. When *P* < .1 or *I*2 > 50% indicated statistical heterogeneity, a random effects model was used to calculate the outcomes; otherwise, the fixed effect model was considered.^[29]^

### 2.8 Subgroup analysis and assessment of heterogeneity

If there was high heterogeneity in the studies, we performed subgroup analysis to explore the differences in age, gender, interventions, and course of disease/treatment.

We used funnel plots to identify whether there was small study bias if 10 or more studies were included. The asymmetry of funnel plots suggests the possibility of small study effects, and the results of analysis were explained cautiously.

### 2.9 Sensitivity analysis

Sensitivity analysis will be performed to test the robustness of findings if there are sufficient studies included. We will conduct sensitivity analysis by excluding

(1) studies with high risks of bias
(2) outliers that are numerically distant from the rest of the data.

### 2.10 Confidence in cumulative evidence

In this study, the level of evidence on outcomes will be assessed using an approach based on the Grades of Recommendations Assessment, Development and Evaluation. The quality of the body of evidence will be assessed based on 5 factors, including study limitations, effect consistency, imprecision, indirectness, and publication bias. The assessments will be categorized as high, moderate, low, and very low quality.

### 2.11 Ethics and Dissemination plans

Ethical approval is not required as this protocol is for a systematic review. In this study, participants are not recruited and data are not collected from participants. The review will be disseminated through peer-reviewed publications.

## 3 Discussion

COPD patients are more likely to be infected with influenza virus, which leads to acute exacerbation. However, the application of SFJDC has great benefits in the treatment of viral infection, and can be used as a combination therapy to overcome the adverse reactions of Western antiviral drugs. Some studies think that Shufeng Jiedu Capsule (SFJDC) not only had inhibitory effects on viral proliferation and anti-inflammation, but also exhibited certain immunoregulatory functions.^[11]^

Moreover, Yao and colleagues demonstrated that SFJDC alleviated clinical symptoms of AECOPD patients and shortened their length of hospital stay.^[30]^ As a supplementary and alternative medicine for influenza prevention and treatment, what's more important is SFJDC has reduced the social and economic burden, especially in developing countries.^[31]^ Therefore, the combination of SFJDC and Western antiviral drugs is expected to make a breakthrough in the theoretical and clinical application of respiratory diseases, especially AECOPD patients.

To our knowledge, this is the latest systematic review of evidence based on more RCTs for SFJD in the treatment of AECOPD. We hope the results of this study could provide a reference for the treatment of AECOPD patient.

## Author contributions

The study was conceptualized by HJR and SYW. The search strategy was developed by SYW, YHJ, and YRW. The protocol was drafted by HJR. HJR and SYW revised the manuscript. HJR submitted the manuscript for publication. All authors have read and approved the final manuscript.

**Conceptualization**: Huijun Ren and Shiyu Wang.

**Funding acquisition**: Shiyu Wang

**Methodology**: Huijun Ren, Yuhao Jiang, Yirong Wang, Jingying Wang

**Supervision**: Shiyu Wang

**Writing – original draft**: Huijun Ren.

**Writing – review & editing**: Huijun Ren
